# IL-33 Inhibits Hepatitis B Virus through Its Receptor ST2 in Hydrodynamic HBV Mouse Model

**DOI:** 10.1155/2020/1403163

**Published:** 2020-04-28

**Authors:** Xiuzhu Gao, Xiumei Chi, Xiaomei Wang, Ruihong Wu, Hongqin Xu, MengRu Zhan, Dong Li, Yanhua Ding, Damo Xu, Junqi Niu

**Affiliations:** ^1^Department of Hepatology, The First Hospital of Jilin University, Jilin University, 71 XinMin Street, Changchun, Jilin Province 130021, China; ^2^Phase I Clinical Research Center, The First Hospital of Jilin University, Jilin University, 71 XinMin Street, Changchun, Jilin Province 130021, China; ^3^Department of Immunology, College of Basic Medical Sciences, Jilin University, Changchun 130021, China; ^4^Medical School, Shenzhen University, Shenzhen 518052, China

## Abstract

Interleukin-33 has been demonstrated to be associated with liver damage. However, its potential value in hepatitis B virus (HBV) infection remains unknown. This study was designed to investigate the role of IL-33 in hydrodynamic HBV mouse model. Different doses of IL-33 were used to treat HBV wild-type, ST2 knockout, CD8+ T depletion, NK depletion C57BL/6 mice and C.B-17 SCID and nod SCID mouse, respectively. The concentrations of HBV DNA, HBsAg, HBeAg, and molecules related to liver function were detected in the collected serum at different time points from model mice. Intrahepatic HBcAg was visualized by immunohistochemical staining of liver tissues. *In vitro*, hepG2 cells were transfected with pAAV-HBV 1.2, then treated with IL-33. The results showed that IL-33 significantly reduced HBV DNA and HBsAg in a dose-dependent manner in HBV wild-type mice. However, in the IL-33 specific receptor ST2 knockout mice, their antiviral effects could not be exerted. Through immunodeficient animal models and *in vivo* immune cell depletion mouse model, we found that IL-33 could not play antiviral effects without NK cells. Moreover, IL-33 could reduce the levels of HBsAg and HBeAg in the supernatant of HBV-transfected hepG2 cells *in vitro*. Our study revealed that IL-33 could inhibit HBV through ST2 receptor in the HBV mouse model, and this effect can be impaired without NK cell. Additionally, IL-33 had the direct anti-HBV effect *in vitro*, indicating that IL-33 could be a potent inducer of HBV clearance and a promising drug candidate.

## 1. Introduction

Chronic hepatitis B is a global public health problem. The current anti-HBV drugs are mainly nucleoside analogs and interferon; nucleoside analogs only inhibit the replication of the virus but cannot clear the hepatitis B virus and interferon has severe side effects. Therefore, it is a research hotspot to find new mechanism and develop medication with strong antiviral effect and less side effects, which can cure hepatitis B without liver damage.

Interleukin-33 (IL-33), a member of the IL-1 family, binds to its receptor carcinogenic suppressor 2 (ST2) [1]. IL-33 is mainly derived from endothelial cells and epithelial cells [2]; however, fibroblasts, monocytes, macrophages, dendritic cells, and hepatocytes can also express IL-33. Necrotic cells have been shown to release the biologically active precursor IL-33 as an “alarmin” [3]. Therefore, IL-33 can induce a variety of downstream immune responses when it is released from cells and is considered to play a key role in infectious and inflammatory diseases. Studies have confirmed that IL-33 plays an important role in HBV-related diseases. The concentrations of serum IL-33 in CHB patients are significantly higher than those in the healthy controls [4]. Serum levels of IL-33 and soluble ST2 vary in different courses of chronic hepatitis B virus infection, and the serum levels of IL-33 and soluble ST2 elevate as serum ALT levels increased in patients with CHB [5]. IL-33 can also induce clearance of HBV in a mouse model [6]. Previous findings suggest that IL-33 may activate follicular helper T cells, leading to the promotion of humoral responses to HBV during the pathogenic process [7]. Interestingly, it has been proved that the IL-33/ST2 axis downregulated concanavalin A-induced liver injury [8] and IL-33 has direct protective effects on hepatocytes, associated with the activation of NF-*κ*B, p38 MAPK, cyclin D1, and Bcl-2 that limits liver injury in mice [9]. These data reveal that IL-33 is a potent regulator of HBV clearance and valid drug candidates.

As a part of innate immunity, natural killer (NK) cells form the first and most important line of defense against pathogenic microorganisms due to their cytotoxicity and secretion of cytokines. The role of IL-33 in promoting type 2 immune responses and tissue inflammation has been well established. However, the relationship of IL-33 and NK cells in hepatitis B virus infection is still limited. The imbalance of NK cell receptors and suppressed NK cell function have been demonstrated in patients with chronic hepatitis B [10]. Decreased activating receptors and increased inhibitory receptors expressed on NK cells, accompanied by their defective functions, have been observed in chronic hepatitis B patients [11, 12]. It has been reported that NK cells are able to sense HBV infection during the early stage or immune-clearance stage of HBV infection, which probably contributes to contain the HBV [13]. The observations in HBV mice reveal that NK cell-derived antiviral cytokines may be responsible for early viral clearance during HBV infection [14]. These findings suggest that NK cell may play a vital role in early viral clearance during HBV infection.

In this study, we tested the hypothesis that IL-33 inhibited hepatitis B virus through its receptor ST2 in hydrodynamic HBV mouse model. We used wild-type, ST2 knockout, CD8+ T depletion, NK depletion C57BL/6 mice and C.B-17 SCID and nod SCID mouse to establish the HBV mouse model, respectively, and then used different doses of IL-33 to treat HBV mouse. Additionally, IL-33 had the direct anti-HBV effect *in vitro*. Our findings indicated that IL-33 could reduce HBV through its receptor ST2, but not damage liver function, which showed IL-33 as a potent inducer of HBV clearance and a promising drug candidate.

## 2. Materials and Methods

### 2.1. HBV Mouse Model

C57BL/6, C.B-17 SCID, nod SCID mice (male, 6–8 weeks old, from Charles River Laboratories, Beijing) and ST2 knockout mice (kindly provided by Professor Weihua Xiao, Institute of Immunology, University of Science and Technology of China) were heavily anesthetized by intraperitoneal injection (IP) of 100 mg/kg ketamine hydrochloride, 20 mg/kg xylazine; for a 20 g mouse, inject 0.2 ml xylazine/ketamine cocktail. 10 mg of pAAV-HBV 1.2 (kindly provided by Professor Xinwen Chen, Wuhan Institute of Virology, Chinese Academy of Sciences) was injected into the tail veins of mice in a volume of PBS equivalent to 8% of the mouse body weight. The total volume was delivered within 5 seconds [15]. The serum specimens were assayed for quantitative HBsAg, HBeAg, HBV DNA, and ALT and AST at the indicated times after injection. At the endpoint, animals were placed in a box that was completely filled with carbon dioxide until they lost consciousness, and then, mice were sacrificed with their necks broken without pain. The livers of mice were preserved in formaldehyde solution for immunohistochemical analysis. The experimental protocols for animal studies were established in accordance with the recommendations in the Guide for the Care and Use of Laboratory Animals of the National Institutes of Health. The experimental protocols were approved by the Animal Research and Protection Committee of Jilin University (Changchun, China), and the project approval number is 2017-162.

### 2.2. NK and CD8+ T Cell Depletion

For NK and CD8+ T cell depletion in C57BL/6 mice, 300 *μ*g anti-NK1.1 (BioXCell) and anti-CD8 (BioXCell) were injected intraperitoneally at days 0, 2, 4, and 6, respectively. Depletion was verified by flow cytometry.

### 2.3. Immunohistochemistry

Liver tissues were collected from mice killed at the indicated time points. Intrahepatic HBcAg was visualized by immunohistochemical staining of tissues embedded in paraffin by rabbit anti-HBc antibodies (DAKO, Carpinteria, CA; Biomeda, Foster City, CA) and Envision System, HRP (diaminobenzidine) (DAKO).

### 2.4. Flow Cytometry

The depletion of NK cells and CD8+ T cell was verified by FACS. Briefly, whole blood was stained in with PerCP Cy5.5-anti-NK1.1 (BD Pharmingen), PerCP-anti-CD8 (BD Pharmingen), FITC-anti-CD3 (BD Pharmingen), and PerCP-anti-CD4 (BD Pharmingen) at room temperature for 30 min. Red cells were lysed by a lysing solution (BD Pharmingen). After being washed with PBS 2 times, the frequency of NK cells and CD8+ T cells was determined by flow cytometric analysis on the FACS Calibur (BD, San Jose, CA) using FlowJo software analysis.

### 2.5. Serological Analysis of Hepatitis

The levels of HBsAg and HBeAg were measured using Roche COBAS 411 Immunoassay System (Roche Diagnostics, Grenzach, Germany). Serum HBV DNA levels were measured using the COBAS AmpliPrep/COBAS TaqMan assay (Roche Molecular Diagnostics, Grenzach, Germany) with a 15 U/ml lowest detection limits. The levels of serum ALT and AST and cholinesterase were detected using a Biochemistry Automatic Analyzer (Roche Diagnostics).

### 2.6. Cell Culture

Human HepG2 cells were cultured in Dulbecco's modified Eagle medium (DMEM) supplemented with 10% fetal bovine serum, 100 IU/ml penicillin, and 100 *μ*g/ml streptomycin. Cells were cultured in an incubator at 37°C with 5% CO_2_ and 100% humidity. Cell culture medium was changed every 3 days.

### 2.7. Transfection of pAAV-HBV 1.2 and Treatment with IL-33

pAAV-HBV 1.2 (300 *μ*g/ml) was transfected into HepG2 cells with LyoVec transfection reagent. The cell cultures were replaced with fresh culture medium containing 0, 1, and 10 ng/ml of IL-33 4 h posttransfection. After 24 and 48 hours, supernatant from each group was independently collected for determination of HBsAg and HBeAg.

### 2.8. Statistical Analysis

Statistics were performed using Prism statistical software (GraphPad). All analyses were unpaired, one-way, nonparametric Mann–Whitney *U* tests.

## 3. Results

### 3.1. IL-33 Inhibits Hepatitis B Virus Replication in Hydrodynamic HBV Mice with Hepatic Protective Effect

**Figure 1:**
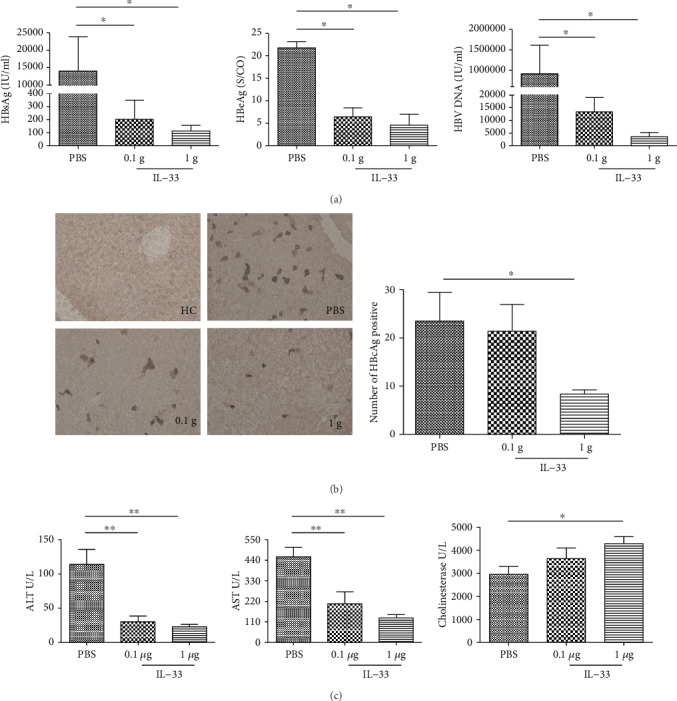
IL-33 treatment inhibits HBV in the hydrodynamic HBV mouse model in a dose-dependent manner. C57BL/6 mice were injected intraperitoneally with IL-33 (0.1 and 1 *μ*g/mouse) and PBS (1 ml/mouse) daily for 1 week, respectively. The concentrations of HBsAg, HBeAg, and HBV DNA in the serum of HBV mice (a). Images of HBcAg staining detected by histological study in liver sections were from HBV mice (b). The levels of ALT and AST and cholinesterase in the serum HBV mice (c). The statistical significance of the data was determined using the Mann–Whitney test; *P* value is shown in each test.

To elucidate the potential role of IL-33 in hydrodynamic HBV mouse model, after the establishment of the HBV mouse model, mice were treated i.p. with IL-33 (0.1 *μ*g and 1 *μ*g) or PBS daily for 6 days. As shown in Figure 1, the levels of serum HBsAg, HBeAg, and HBV DNA (Figures 1(a)–1(c)) in the IL-33-treated mice were significantly lower than those in the PBS-injected mice in a dose-dependent manner (*P* < 0.05, respectively). In parallel, similar patterns of HBcAg were detected in the liver of the IL-33-treated and PBS-treated mice (Figure 1(d)). More remarkable, the levels of serum ALT and AST in IL-33-treated mice were significantly lower than those in the PBS-injected mice in a dose-dependent manner (*P* < 0.05, respectively), while serum cholinesterase in IL-33-treated mice was higher (Figure 1(e)). These data clearly demonstrated that treatment with IL-33 significantly reduced HBV virus loads and HBV-related antigens in hydrodynamic HBV mice without liver damage.

### 3.2. IL-33 Cannot Exert Anti-HBV Effect in ST2 Knockout Mice

**Figure 2:**
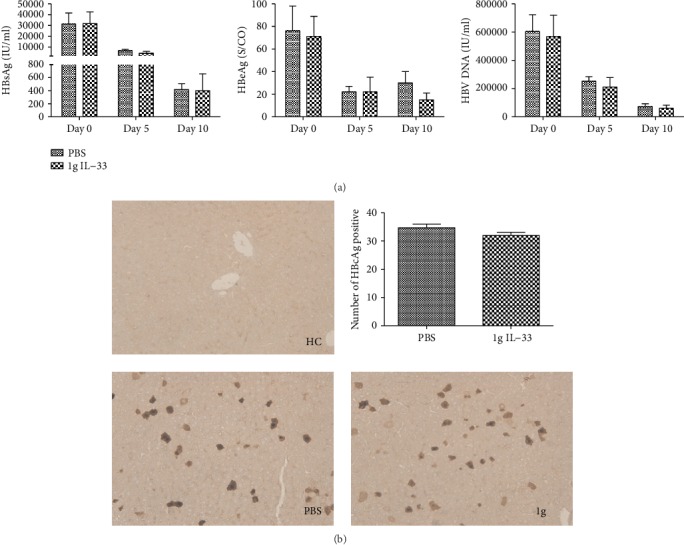
IL-33 has no antiviral effect in ST2 knockout mice. The concentrations of serum HBsAg, HBeAg, and HBV DNA in HBV ST2 knockout mice (a). Images of HBcAg staining were detected by histological study in liver sections from HBV ST2 knockout mice (b). The statistical significance of the data was determined using the Mann–Whitney test; *P* value is shown in each test.

IL-33 exerts its cytokine activity by binding to a heterodimer formed by its specific primary receptor ST2. To elucidate whether IL-33 plays a role without its receptor ST2, ST2 knockout mice were used to establish a HBV mouse model and treated with IL-33. As shown in Figure 2, there were no differences of serum HBsAg, HBeAg, and HBV DNA (Figures 2(a)–2(c)) between IL-33- and PBS-injected mice. And similar patterns of HBcAg were detected in the liver of the IL-33- and PBS-injected mice (Figure 2(d)). These data proved that IL-33 could not exert anti-HBV effect without ST2.

### 3.3. The Anti-HBV Effect of IL-33 Has Been Impaired in NK Depletion HBV Mice

**Figure 3:**
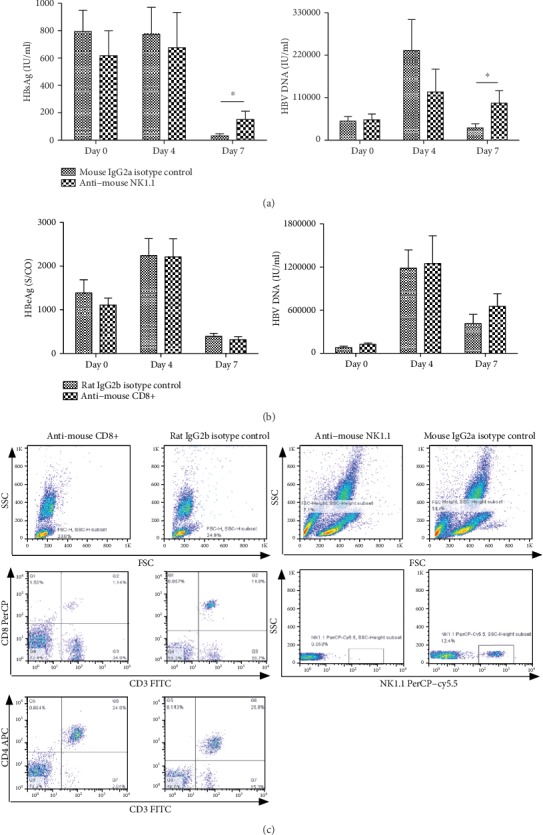
The anti-HBV effect of IL-33 has been impaired in NK depletion HBV mice. After the treatment of IL-33, the levels of HBsAg and HBV DNA in NK depletion HBV mice are higher than its counterparts in WT HBV mice (*P* = 0.026 and *P* = 0.05, respectively) (a). However, there is no difference in the levels of serum HBsAg and HBV DNA between the WT HBV mice and CD8+ T cell depletion HBV mice after the treatment of IL-33 (b). The depletion of NK cells and CD8+ T cells was verified by FACS; compared with their isotype controls, NK cells and CD8+ T cells were almost undetectable (c). The statistical significance of the data was determined using the Mann–Whitney test; *P* value is shown in each test.

300 *μ*g anti-NK1.1 and anti-CD8 (BioXCell) were injected intraperitoneally at days 0, 2, 4, and 6, respectively. NK and CD8+ T cell depletion in C57BL/6 mice was verified by FACS. After the depletion of NK and CD8+ T cell, HDI HBV mouse model was established. Then, the HBV mice were treated with IL-33 (1 *μ*g). The results show that there is no difference in the levels of HBsAg, HBeAg, and HBV DNA between the WT HBV mice and CD8+ T cell depletion HBV mice after the treatment of IL-33 (Figures 3(a)–3(c)). However, compared with WT HBV mice, the levels of HBsAg and HBV DNA in NK depletion HBV mice are higher (*P* = 0.026 and *P* = 0.05, respectively) (Figures 3(d) and 3(f)). And there is no difference in HBeAg between the two groups (Figure 3(e)).

### 3.4. IL-33 Partly Inhibits the Replication of HBV in Immunodeficient Mice

**Figure 4:**
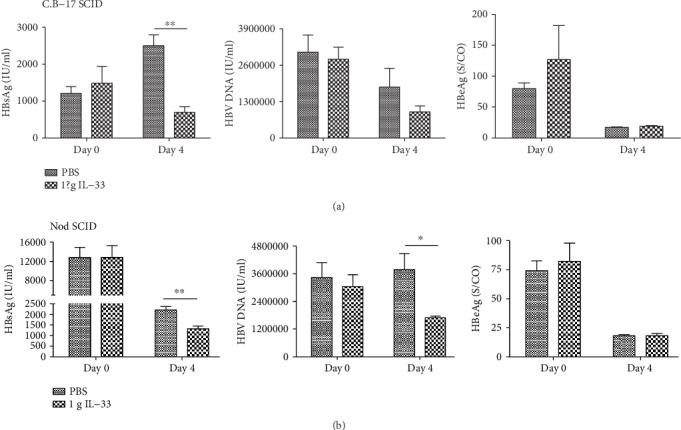
IL-33 can reduce the concentration of HBsAg, but has no significant effect on HBV DNA in C.B-17 SCID HBV mice (a). IL-33 can reduce the concentration of HBsAg and HBV DNA in nod SCID HBV mice after IL-33 treatment (b). IL-33 has no effect on HBeAg in both C.B-17 SCID and nod SCID HBV mice. The statistical significance of the data was determined using the Mann–Whitney test; *P* value is shown in each test.

We use C.B-17 SCID and nod SCID mouse to establish the HBV mouse model, respectively, and then use IL-33 to treat HBV mouse for 6 days. The results show that IL-33 can reduce the concentration of HBsAg (Figure 4(a)), but has no effect on HBeAg and HBV DNA (Figures 4(b) and 4(c)) in C.B-17 SCID HBV mice. Apart from that, for nod SCID HBV mice, IL-33 can reduce the concentration of HBsAg (Figure 4(d)) and HBV DNA (Figure 4(f)) after 3 days of IL-33 treatment, but until 6 days, there is no difference between the two groups. Consistent with the results in C.B-17 SCID and NK depletion HBV mice, IL-33 has no effect on HBeAg (Figure 4(e)).

### 3.5. IL-33 Inhibits the Repletion of HBV *In Vitro*

**Figure 5:**
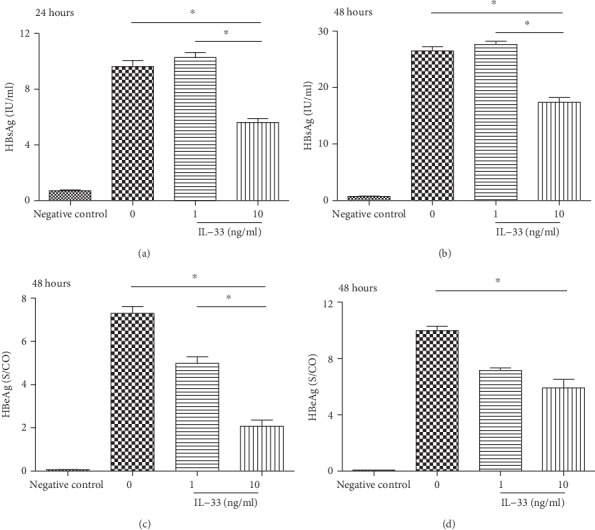
Effect of IL-33 on the secretion of HBsAg and HBeAg in the HepG2 cell line transfected with pAAV-HBV 1.2. After treatment with different concentrations of IL-33 (0, 1, and 10 ng/ml) for 24 and 48 hours, HBsAg in the supernatant decreased in a dose-independent manner (a, b); HBeAg in the supernatant significantly decreased in a dose-independent manner (c, d). The statistical significance of the data was determined using the Mann–Whitney test; *P* value is shown in each test.

IL-33 has the direct anti-HBV effect in vitro. The pAAV-HBV 1.2 (300 *μ*g/ml) was transfected into HepG2 cells. After treatment with different concentrations of IL-33 (0, 1, and 10 ng/ml) at different time points (24 and 48 hours), HBsAg and HBeAg (Figure 5) secreted into the medium were reduced in a dose-dependent manner.

## 4. Discussion

The current anti-HBV drugs are mainly nucleoside analogs. However, nucleoside analogs require long-term or even life-long medications. And nucleoside analogs only inhibit the replication of the virus and cannot clear the hepatitis B virus. Therefore, it is a research hotspot for antihepatitis B treatment to find new drugs with strong antiviral effect and low side effects, which can cure chronic hepatitis B virus infection. As a member of the IL-1 family, IL-33 was originally described as an inducer of type 2 immune responses, activating TH2 cells and mast cells [1]. But subsequent studies show that IL-33 can activate leukocytes including TH1 cells, Treg cells, ILC2s, CD8+ T cells, and NK cells by its receptor ST2 [16–20]. Thus, IL-33 can play an important role in innate and adaptive immunity, contributing to tissue homeostasis and responses to environmental stresses.

Our previous work found that, compared with healthy controls, serum levels of IL-33 and its soluble receptor ST2 were elevated in patients infected with chronic hepatitis B. In addition, *in vitro* experiments showed that IL-33 can reduce the secretion of HBsAg, HBeAg, and HBV DNA of HepG2.2.15, and the effect was in a dose-dependent manner [4]. Another study found that the levels of IL-33 and ST2 elevated accompanied by increased ALT in patients with CHB [5]; they might indicate liver damage for patients with CHB which was conformed to its “alarmin” effect. Recent studies reported that IL-33 was associated with HBV clearance in mice [6, 7]. However, the role of IL-33 in innate immunity is still unclear.

HBV cannot infect mouse hepatocytes; consequently, transfection-based mouse models of HBV including HDI mice do not reflect full viral life cycle completely. Apart from that, high volume of intravenous tail injection can lead to transient liver damage. Nevertheless, these models enable analysis and comparison of virus-host interactions in mice and human. In this study, we use a hydrodynamic HBV mouse model to mimic chronic hepatitis B infection in humans. The results show that IL-33 inhibits hepatitis B virus replication in the hydrodynamic HBV mouse model with hepatic protective effect. However, IL-33 cannot exert anti-HBV effect in ST2 knockout mice. And then, we use IL-33 to treat CD8+ T depletion and NK depletion HBV mice to reveal which immunity is involved in this process. As a result, after the treatment of IL-33, there is no difference between wild-type and CD8+ T depletion HBV C57BL/6 mice, while compared with its counterparts, NK depletion HBV mice have higher serum HBsAg and HBV DNA. The results above show that NK cells may play an antiviral role in this process. Then, we use C.B-17 SCID and nod SCID mouse to verify this result. C.B-17 SCID mouse exhibits severe immunodeficiency symptoms, lacking B cell and T lymphocyte while having normal NK cells, macrophages, and granulocytes. The results show that compared with the PBS control groups, IL-33 can reduce the level of HBsAg in the serum of HBV C.B-17 SCID mice. But IL-33 exerts no effect on HBeAg and HBV DNA. Interestingly, NOD SCID for the SCID mutation has impaired T and B cell lymphocyte development, which additionally results in deficient NK cell function. Our study shows that IL-33 can reduce the concentration of HBsAg and HBV DNA in nod SCID HBV mice after IL-33 treatment, indicating that IL-33 can play its anti-HBV role through other cells. It has been proved that the major targets of IL-33 *in vivo* are tissue-resident immune cells, and mast cell is one of them [21]. IL-33 is a member of the IL-1 family capable of inducing mast cell responses and enhancing IgE-mediated activation [22]. Apart from that, studies have uncovered important roles of IL-33 in the activation of immune cells such as eosinophils, neutrophils, and macrophages. IL-33 restricts tumor growth and inhibits pulmonary metastasis in melanoma-bearing mice through eosinophils [23]. IL-33 also drives eosinophil infiltration and pathogenic type 2 helper T cell immune responses leading to chronic experimental ileitis [24]. However, so far, there are seldom evidences about these cells above in HBV infection.

Meanwhile, a recent study demonstrated that IL-33 signaled through CD8+ T cells [25] and a study evidenced that a chronic HBV replication mouse model conducted by hydrodynamic injection of pAAV/HBV1.2 plasmid into C57BL/6 mice does not induce an active NK cell response [26]. The discrepancy might be due to the difference in the time of experiment. Antigen-specific CD8 T cells are needed at least 10 days to be differentiated and moved to the inflamed tissue. In our case, the mice were only treated with IL-33 for 5 days; at this short period, the antigen-specific CD8+ T cells were unlikely to be expended and detected. Additionally, our model was an acute infection model and innate immune cells, including NK cells, played a critical role. Some researchers used chronic animal model, and therefore, adaptive rather than innate cells were more important.

Additionally, it has been reported that the IL-33-deficient mice exhibited more severe ConA liver injury than WT controls [27]. Additionally, IL-33 has direct protective effects on hepatocytes, associated with the activation of NF-*κ*B, p38 MAPK, cyclin D1, and Bcl-2 that limits liver injury [9]. As mentioned above, IL-33 plays a protective role in the resolution of inflammation and repair of liver damage. This may be due to the ability of IL-33 to target ST2 expressed on Treg cells and ILC2s [18]. In our study, we found that IL-33 has the direct anti-HBV effect on HBV-transfected hepG2 cells *in vitro* (Figure 5). Moreover, IL-33 can inhibit the replication of HBV without liver damage, which further validates the protective effect of IL-33. However, the mechanism still needs to be further investigated.

The liver is considered to be a unique immune organ based on innate immunity, due to its unusually large number of innate immune cells, especially NK cells. Tumoral expression of IL-33 exerts its antitumor effects through CD8+ T cells and NK cells [28]. IL-33/ST2 signaling was related to the development of several types of tumors and with impaired cytotoxicity of NK cells [29]. Although there are good evidences showing that IL-33 can kill tumor in mice via NK cells [28, 30], the role of IL-33 in hepatocellular carcinoma is still controversial depending on how IL-33 is applied. Our study reveals that IL-33 can inhibit HBV through its ST2 receptor in the HBV mouse model, and this antiviral effect has been impaired without the NK cell. However, whether and how IL-33 can activate NK cells are still unknown. Additionally, future investigation still needs to address how IL-33 exerts anti-HBV effect without hepatocyte damage.

## 5. Conclusions

Our study revealed that IL-33 inhibits hepatitis B virus through its receptor ST2 in the hydrodynamic HBV mouse model, and this effect can be impaired without the NK cell. Additionally, IL-33 had the direct anti-HBV effect *in vitro*, indicating that IL-33 could be a potent inducer of HBV clearance and a promising drug candidate.

## Data Availability

The [DATA TYPE] data used to support the findings of this study are included within the article.
